# Analysis of gut microbiota in Restless Legs Syndrome: searching for a metagenomic signature

**DOI:** 10.1093/sleep/zsaf383

**Published:** 2025-12-11

**Authors:** Angelica Montini, Camilla Pellegrini, Giuseppe Loddo, Francesco Ravaioli, Luca Baldelli, Greta Mainieri, Chiara Pirazzini, Elena Mazzotta, Francesco Carano, Claudia Sala, Sara De Fanti, Maria Giulia Bacalini, Federica Provini

**Affiliations:** Department of Biomedical and NeuroMotor Sciences, University of Bologna, Bologna, Italy; IRCCS Istituto delle Scienze Neurologiche di Bologna, Bologna, Italy; Azienda AUSL di Bologna, Department of Primary Care, Bologna, Italy; IRCCS Istituto delle Scienze Neurologiche di Bologna, Bologna, Italy; Department of Biomedical and NeuroMotor Sciences, University of Bologna, Bologna, Italy; IRCCS Istituto delle Scienze Neurologiche di Bologna, Bologna, Italy; IRCCS Istituto delle Scienze Neurologiche di Bologna, Bologna, Italy; Department of Medical and Surgical Sciences, University of Bologna, Bologna, Italy; Department of Biomedical and NeuroMotor Sciences, University of Bologna, Bologna, Italy; Department of Rare Skeletal Disorders, IRCCS Istituto Ortopedico Rizzoli, Bologna, Italy; Department of Medical and Surgical Sciences, University of Bologna, Bologna, Italy; IRCCS Istituto delle Scienze Neurologiche di Bologna, Bologna, Italy; Department of Biomedical and NeuroMotor Sciences, University of Bologna, Bologna, Italy; Department of Biomedical and NeuroMotor Sciences, University of Bologna, Bologna, Italy; IRCCS Istituto delle Scienze Neurologiche di Bologna, Bologna, Italy

**Keywords:** Restless Legs Syndrome; Restless Legs Syndrome—neurobiology, Restless Legs Syndrome—clinical assessment, insomnia, neurological disorders, sleep-related movement disorders, gut microbiota, metagenomics, brain–gut–microbiome axis, inflammation

## Abstract

**Study Objectives:**

We aim to analyze the microbiota composition in Restless Legs Syndrome (RLS) patients and its relationship with the different RLS phenotypes.

**Methods:**

We recruited idiopathic RLS (*RLS*) and insomnia (*INS*) patients and healthy subjects (*CTRL*). Validated questionnaires (Pittsburg Sleep Quality Index, International Restless Legs Syndrome Study Group Rating Scale, Insomnia Severity Index, Beck Depression Inventory-II) were administered in the *RLS* and *INS*. Fecal microbiota was analyzed by 16S rRNA gene sequencing according to Illumina metagenomics standard procedure on MiSeq Platform. Dada2 pipeline was used to process sequencing data, while DESeq2 and Aldex2 tools were used to calculate differential abundance taxa, correcting for age, sex, body mass index, sequencing run, and presence of mood disorders.

**Results:**

The sample included 37 *RLS* (28 females, mean age 64.78 years), 31 *INS* (22 females, mean age 60.64 years), and *33 CTRL* (24 females, mean age 62.54 years). Differential abundance analysis revealed a statistically significant decrease in the abundance of *Lachnoclostridium* and *Flavonifractor* genera in *RLS* compared to *CTRL* and *INS*, but not in the *INS* compared to *CTRL. Lachnoclostridium* abundance tended to decrease with long disease duration and a predominant motor phenotype. In the RLS group, several genera were identified as significantly associated with International Restless Legs Syndrome Study Group Rating Scale and Pittsburg Sleep Quality Index scores.

**Conclusions:**

Although only a few previous studies have reported the presence of small intestinal bacterial overgrowth in RLS, to the best of our knowledge this is the first study to highlight significant differences in the gut microbiota composition of *RLS* compared to both *CTRL* and *INS*, identifying a specific RLS metagenomic signature.

**Statement of Significance**

This is the first study to comprehensively characterize the gut microbiota in patients with Restless Legs Syndrome (RLS), identifying a distinct microbial signature compared to insomnia patients and healthy controls. We observed alterations alpha and beta diversity and specific changes in bacterial families and genera, some of which significantly correlated with RLS clinical features. In particular, *Lachnoclostridium* genus was significantly reduced in *RLS* and tended to be less abundant in patients with longer disease duration and without sensory symptoms. This genus is known to modulate systemic inflammation through the production of short-chain fatty acids, suggesting a potential link between gut dysbiosis, inflammation, and dopaminergic dysfunction. These findings support a role for gut microbiota alterations in RLS pathogenesis and open new avenues for microbiota-based diagnostic and therapeutic strategies.

## Graphical Abstract

**Graphical Abstract:**
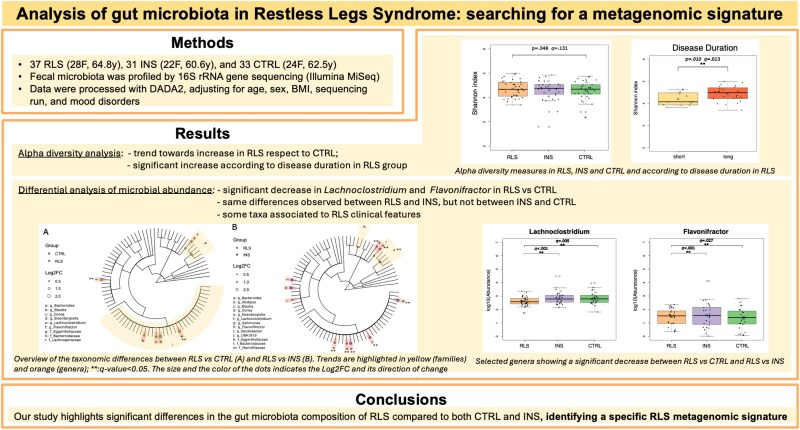

## Introduction

Restless Legs Syndrome (RLS) is characterized by an irresistible urge to move the legs, accompanied or not by sensory symptoms, during rest, especially in the evening and at night [1]. RLS affects up to 10 per cent of adults [2] and may present a broad range of clinical manifestations, leading to sleep disruption and significant impairment of patients’ daytime functioning and overall quality of life [3]. The diagnosis of RLS remains challenging due to the possible presence of several mimics. A better understanding of RLS pathogenesis could provide clinicians with supportive diagnostic tools and pave the way for new effective therapeutic approaches. In fact, despite progress in moving beyond the classical classification of RLS into primary and secondary forms—the latter including those associated with iron deficiency and renal insufficiency—RLS pathogenesis still remains incompletely understood. A multifactorial model involving gene–environment interactions could explain the wide RLS clinical spectrum [4]. Recently, multi-omics have been shown to be promising tools for investigating both predisposing and precipitating factors for the RLS symptoms. Genomic, proteomic, and transcriptomic studies have confirmed the involvement of iron metabolism in the pathogenesis of RLS, while also highlighting the contribution of emerging factors such as inflammation [5,6]. In fact, inflammatory conditions, such as infectious and autoimmune disorders, could trigger or exacerbate RLS symptoms (e.g. by inducing iron deficiency through hepcidin upregulation) [7]. Moreover, RLS is frequently associated with increased sleep latency and sleep fragmentation, due to arousals and Periodic Limb Movements, which could cause inflammation and immunological changes [8,9]. Data regarding serum/plasma C-reactive protein levels are controversial, but other cytokines (IL-6, TNF-α, IL-1β, IL-17) increase in RLS patients and could represent promising biomarkers [10]. These cytokines may induce RLS symptoms by dysregulating dopamine receptor functions [11,12].

Sleep and the microbiota share a bidirectional relationship, potentially mediated by the brain–gut–microbiome axis, as confirmed by human and animal models [13]. Acute and chronic sleep fragmentation and deprivation have been shown to alter the gut microbial composition [14–16], while probiotics could mitigate risks associated with sleep deprivation [17]. Metagenomic studies in insomnia (INS) patients have identified a bacterial signature, characterized by a reduced abundance of *Faecalibacterium* and an increased abundance of *Blautia* genera, which is significantly associated with higher Pittsburg Sleep Quality Index (PSQI) scores and elevated plasma levels of IL-1β [18]. In 2011, Weinstock and Walters [19] described the presence of small intestinal bacterial overgrowth (SIBO) in 69 per cent of RLS patients compared to 28 per cent of general population controls, using the breath test. This condition was often associated with Irritable Bowel Syndrome (IBS) in RLS groups but also showed an independent association with RLS. More recently, Blum et al. [20], in a series of seven RLS patients, confirmed that this is a condition associated with gut dysbiosis. However, specific microbiota analyses in RLS patients are still lacking in the literature.

The mechanism underlying the interaction between the gut microbiome and the central nervous system (CNS) remains incompletely understood. However, previous studies have shown that gut bacteria produce metabolites capable of influencing various pathways, including the nervous [21], endocrine [22], and immune systems [23] while at the same time regulating the permeability of both the gut and the blood–brain barrier [24]. Furthermore, the gut microbiota directly influences CNS functioning by producing peripheral neurotransmitters such as dopamine, GABA, and serotonin, which are sensed by the vagus nerve and facilitate communication between the CNS and the enteric nervous system [25].

Hence, our study aimed to explore whether the microbiota is implicated in the pathophysiology of RLS, serving as a microenvironmental factor that could trigger the onset of RLS symptoms or influence the RLS phenotype. To achieve this, we compared the fecal microbial communities of RLS patients with those of healthy individuals and a group of patients with chronic INS, given the high prevalence of INS among RLS patients. Lastly, we aimed to investigate whether changes in microbiota parameters correlate with the clinical features of RLS.

## Materials and Methods

### Subjects

Patients with Restless Leg Syndrome (*RLS*) attending the IRCCS-ISNB Sleep Center were recruited between December 2022 and February 2024, with diagnoses confirmed by sleep experts (F.P., A.M.) based on the five international diagnostic criteria [1]. Chronic *INS* patients, diagnosed according to the International Classification of Sleep Disorders (ICSD-3-TR) [26] diagnostic criteria, and healthy controls (*CTRL*), matched for sex, and age, were also enrolled. The *CTRL* were recruited from unrelated caregivers of patients or from volunteers in our independent IRCCS-ISNB control cohort. Inclusion criteria for all groups included an age of over 18 years, while exclusion criteria consisted of an ongoing acute inflammatory disease, pregnancy, recent antibiotic use (<1 month), anti-inflammatory drugs, or major sedatives use. Additionally, subjects with concomitant sleep-interfering drugs, sleep disorders, and neurological, severe psychiatric and systemic diseases were excluded from the *CTRL*.

For all groups, physiological, familial, pathological, and pharmacological histories were collected through a semi-structured interview conducted by a sleep expert (A.M.). Specific validated questionnaires for sleep disorders (PSQI; International Restless Legs Syndrome Study Group Rating Scale, IRLS; and Insomnia Severity Index, ISI) and depression (Beck Depression Inventory-II, BDI-II) were administered to the *RLS* and *INS* patients. Comparison of demographic and clinical features among *RLS*, *INS*, and *CTRL* groups was performed using Fisher’s test for two groups and the chi-squared test for three groups. The local Ethical Committee approved the study protocol (CEAVEC 794-2022-SPER-AUSLBO), and informed written consent was obtained from all the participants.

### Sample collection and DNA extraction

Fecal samples were collected in OMNIgene•GUT tubes containing microbial DNA stabilizer (DNA Genotek, Canada). Samples were stored for a maximum of 7 days at room temperature, vortexed 60 s, transferred in cryotubes, and stored at −80°C. Microbial DNA was extracted from 200 μL of defrosted feces using the protocol described by Ghosh et al. [27] with the following modifications: the ASL buffer (QIAGEN, Hilden, Germany) was used to lyse 200 μL of feces; bead-beating was performed inPower Bead Tubes (Ceramic 1.4 mm) using the PowerLyzer 24 Homogenizer (QIAGEN, Hilden, Germany); samples were homogenized two times at 1800 rpm for 3 min, with a 1 min pause between the 2 cycles; after the proteinase K treatment, DNA purification was performed using the QIAamp Fast DNA Stool Mini Kit (QIAGEN, Hilden, Germany). DNA was quantified using Qubit dsDNA Broad Range (BR) Assay Kit (Qiagen, Hilden, Germany) and stored at −20°C before analysis.

### Library construction and sequencing

Library preparation was performed in accordance to Illumina 16S Library Preparation Workflow. Briefly, the hypervariable V3-V4 region of the 16S rRNA gene was amplified with primers 341F and 805. The polymerase chain reaction product was purified using MagSi Prep Plus magnetic beads (Magtivio, NL) andamplified for 8 cycles using Nextera XT Indexed Primer. The final product was purified, quantified using Qubit dsDNA BR Assay Kit (Qiagen, Hilden, Germany), and pooled at equimolar concentration (4 nM). Pooled libraries were denatured with 0.2 N NaOH, diluted, and combined with 10 per cent PhiX Sequencing control V3. The final pool (6 pM) was sequenced on Illumina MiSeq platform using paired-end 2 × 300-bp reads and MiSeq v3 reagents (Illumina, San Diego, CA). Samples were sequenced in two separate runs.

### Bioinformatic processing of 16S rRNA sequencing

Raw paired-end reads were pre-processed according to *DADA2* Big Data pipeline [28] in R (v. 4.2.2). Briefly, for each run, forward and reverse reads were separately checked for quality and trimmed to remove Illumina adapters and low-quality sequences. Filtered reads were deduplicated, merged, and clustered into an amplicon sequence variant (ASV) table. ASVs tables from different runs were then merged together. Finally, after removing chimeric ASVs, taxonomy was assigned against SILVA non-redundant small subunit ribosomal RNA database (v138.1), with default bootstrap confidence value (50 per cent). Samples with low sequencing depth (<20 000 reads) were excluded from the analysis. Taxa that were assigned to neither Bacteria nor Archaea and taxa present in <10 samples (10 per cent of the total sample size) were removed from the ASVs count table.

### Statistical analysis and microbiota composition profiling

All statistical analyses were performed with R (v. 4.2.2). The *phyloseq* package (v 1.42.0) [29] was used for microbial abundance data handling and for calculating alpha and beta diversity at ASVs level. Alpha diversity, an index of the microbial variability within a group, was calculated using several metrics, including the number of observed species, Chao, Shannon, and inverted Simpsons indices. Differential analysis of alpha diversity measurements among *RLS*, *INS*, and *CTRL* groups was performed by ANOVA, using the *Anova* function in the *car* package (v 3.1-2). Differences in alpha diversity metrics with respect to RLS clinical categories—symptoms onset (early, ≤45 years; late, >45 years); disease duration (short, <15 years; long, ≥15 years); presence or absence sensory symptoms [1,30]—were evaluated using ANOVA, while their association with questionnaire scores—IRLS, ISI, and PSQI—were evaluated using linear models. Beta diversity, which measures microbial community dissimilarity among groups, was assessed via PCoA based on Bray-Curtis, weighted and unweighted Unifrac metrics. Differential analysis of inter-individual microbiota diversity across the three groups was performed using the *PERMANOVA* function in *vegan* package (v 2.6-6.1). In addition, the relationship between beta diversity measurements and clinical parameters was evaluated by ANOVA test.

Differences in microbial abundances were evaluated at phylum, family, and genus level. Firstly, dysbiosis was evaluated by calculating the widely accepted index, the Firmicutes/Bacteroidetes ratio, which was compared between groups using ANOVA test. Secondly, differential microbial abundance analyses between *RLS*, *INS*, and *CTRL* were performed according to DESeq2 pipeline [31] using Wald test. In parallel, an alternative analysis of differential abundance was performed using the Aldex2 method [32].

Thirdly, the DESeq2 pipeline was used to assess the relationship between microbial abundance and the previously mentioned RLS clinical parameters, and in addition, the presence or absence of mood disorders, severity of depression according to the BDI-II score, and pharmacological treatment. The scores from the questionnaires were also treated as continuous variables, and the log2 Fold Change value returned by DESeq2 represents the change per unit of the variable under consideration.

Finally, predictive functional profiling was performed using PICRUSt2 (v. 2.6.2). Abundances of predicted functions were normalized with Centered Log Ratio transformation using the *compositions* package (v. 2.0-6). Differential pathway analysis was conducted in R using the *limma* package (v. 3.54.2) comparing RLS to CTR and INS groups.

In all the analyses described above (alpha and beta diversity indices, differential abundance analysis, PICRUSt2 analysis), age, sex, BMI, sequencing batch, and the presence of mood disorders were included as covariates to control for potential confounding effects. Statistical significance was considered at Benjamini-Hochberg (BH) corrected *p*-value (*q*-value) < .05 to account for multiple testing, while nominal *p*-values <.05 were reported as trends in the text.

## Results

### Demographic and clinical features

The demographic and clinical data of the 37 *RLS*, 31 *INS*, and 33 *CTRL* are shown in Table 1. The mean *RLS* disease duration was 26.19 ± 18.28 years; 18 patients (48.65 per cent) presented with early onset (<45 years), including 7 (18.92 per cent) in pediatric age. Sixteen *RLS* (43.24 per cent) had at least one first- or second-degree relative experiencing symptoms consistent with *RLS*. All *RLS* had a chronic course; 27 (78.38 per cent) experienced moderate to severe symptoms according to the IRLS scale, and 18 out of 37 (48.65 per cent) reported sensory symptoms. 12 *RLS* (32.43 per cent) had chronic gastrointestinal diseases that were in remission, and in nine of them the disease involved only the upper digestive tract. In 26 of the 37 *RLS* (70.27 per cent), iron status was assessed at the time of the microbiota evaluation, and in 20 of these (54.05 per cent), ferritin levels were < 75 ng/mL. Additionally, six of these latter patients were taking oral iron at the time of the microbiota analysis. Sixteen *RLS* (43.24 per cent) did not use any drug for treating RLS, while nine patients (24.32 per cent) were under dopaminergic agonists, eight patients (21.62 per cent) were on α2δ-ligands, and only four patients (10.81 per cent) were taking a combination of these two. Additionally, 16 *RLS* were also using medication for INS: 12 (32.43 per cent) were taking benzodiazepines, and 4 (10.81 per cent) were on other treatments (including melatonin, Z-drugs, and daridorexant). *INS* had a mean disease duration of 10.88 ± 13.01 years and a median ISI score of 8.66 ± 6.21 and PSQI score of 9.5 ± 4.9. Fifteen (48.39 per cent) *INS* reported mood disorders and nine (29.03 per cent) suffered from gastrointestinal disorders. Fifteen (48.39 per cent) *INS* did not use any specific drug for treating INS; 4 patients took benzodiazepines, 4 melatonin, 3 Z-drugs, 1 daridorexant; the remaining 4 patients were on a combination of substances (3 took benzodiazepines and melatonin, and 1 took daridorexant and melatonin).

**Table 1:** Demographic and clinical features of restless legs Syndrome patients (*RLS*), Insomnia patients (*INS*), and healthy controls (*CTRL*). Data are presented in mean and standard deviation (SD) or in absolute number (*n*) and percentage (%).

Parameters	RLS (*n* = 37)	INS (*n* = 31)	CTRL (*n* = 33)	*p*-value
Age, years (mean, *SD*)	64.78 ± 12.27	60.64 ± 11.49	62.54 ± 11.42	0.372
Female (*n*, %)	28 (75.67)	24 (70.97)	24 (72.73)	0.905
BMI, kg/m^2^ (mean, *SD*)	27.43 ± 5.68	24.50 ± 3.17	25.86 ± 4.20	0.036
Disease duration, years (mean, *SD*)	26.19 ± 18.28	10.88 ± 13.01	/	<0.001
Sleep questionnaires (mean, *SD*)				
PSQI	13.00 ± 4.74	9.50 ± 4.91	/	0.017
IRLSS	19.21 ± 8.86	/	/	/
ISI	7.06 ± 5.87	8.66 ± 6.21	/	0.372
Comorbidities (*n*, %)				
Mood disorders	22 (59.46)	15 (48.39)	/	<0.001
Anxiety	2 (5.41)	6 (19.35)		
Depression	8 (21.62)	5 (16.13)		
Anxiety and depression	12 (32.43)	4 (12.90)		
Gastrointestinal disorders	12^*^ (32.43)	9^*^ (29.03)	2^*^ (6.06)	0.007
GERD	2 (5.41)	7 (22.58)	2 (6.06)	
Chronic gastritis	8 (21.62)	3 (9.68)	0	
Diverticulosis	0	1 (3.22)	0	
IBS	3 (8.11)	0	0	
RLS treatments (*n*, %)	21 (56.75)	/	/	/
α2δ-ligands	8 (21.62)			
DOPA-agonists	9 (24.32)			
α2δ-ligands + DOPA-agonists	4 (10.81)	16 (51.61)		0.63
INS treatments (*n*, %)	16 (43.24)			
Benzodiazepines	12 (32.43)	4 (12.90)		
Other INS drugs	4 (10.81)	9 (29.03)		
Benzodiazepines + melatonin		3 (9.68)		

α2δ = alpha2delta ligands; DOPA-agonists = dopamine agonists; GERD = Gastroesophageal Reflux Disease; IBS = Irritable Bowel Syndrome; PSQI = Pittsburg Sleep Quality Index; IRLS = International Restless Legs Syndrome Study Group Rating Scale; ISI = Insomnia Severity Index.

^*^Includes patients with multiple gastrointestinal comorbidities; thus, totals may not match the sum of individual conditions. “Other drugs” include melatonin, Z-drugs, and daridorexant.

### Overview of fecal bacterial communities

The 16S rRNA amplicon sequencing yielded a total of 8 852 365 reads (ranging from 24 994 to 181 691 per sample). After prevalence filtering, the dataset was composed of 770 ASVs that were assigned to 7 phyla, 31 families, and 83 genera.

**Figure 1:**
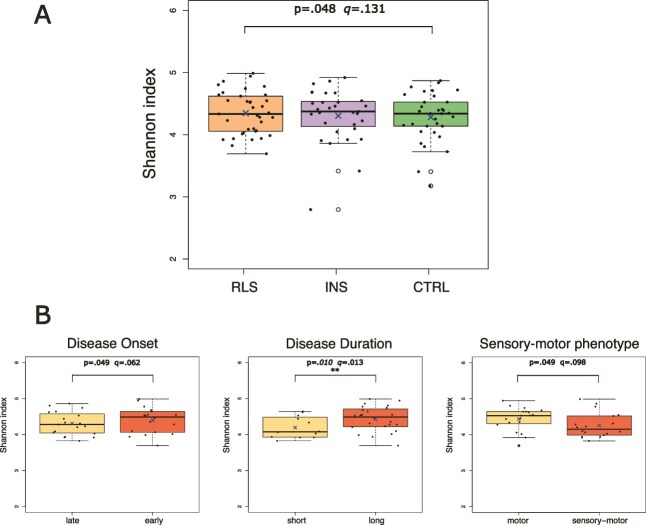
Alpha-diversity analysis. (**Panel A**) Boxplot showing alpha diversity index (Shannon index) in patients with Restless Legs Syndrome patients (*RLS*), insomnia (*INS*), and healthy controls (*CTRL*). Sample size *n* = 101. (**Panel B**) Boxplot illustrating the association of alpha diversity index with *RLS* clinical features: Disease onset (early/late: ≤ 45 vs. >45 years), disease duration (short/long: <15 vs. ≥15 years), and phenotype (motor vs. sensory-motor). Sample size *n* = 37. The central line indicates the median; the *X* indicates the mean; the box represents the interquartile range (IQR), and the whiskers extend to 1.5 × IQR. Black dots represent individual samples; white circles are outliers. Statistical test: ANOVA correcting for age, sex, BMI, sequence batch, and presence of mood disorders. ^**^ indicates BH-adjusted *p*-value (*q*-value) < .05. Reference group in all comparisons is on the left.

No statistically significant (*q*-value < .05) differences in alpha diversity were observed between *RLS* and *CTRL* group, although a trend (nominal *p*-value <.05) toward an increase in *RLS* was found for the Shannon index, which considers both the number of ASVs (richness) and their distribution (evenness) (Figure 1A). Similarly, no statistically significant differences were found between *RLS* and *INS* for any alpha diversity metrics, nor when comparing *INS* to the *CTRL* (Data Set S1). We then assessed the associations between alpha diversity measures and clinical parameters within the *RLS* group. All the indices were statistically significantly higher in patients with longer disease duration; in addition, the Shannon, Chao1, and observed richness indices showed a not significant trend towards an increase in RLS patients with early onset, while the Shannon and Simpson indices tended to be lower in RLS patients with sensory-motor symptoms (Figure 1B and Data Set S2).

The analysis of beta diversity did not show significant (*q*-value < .05) differences between *RLS*, *INS*, and *CTRL* (Data Set S1). When considering clinical features within the *RLS* group, both Unifrac and Bray-Curtis methods were significantly different according to disease duration (*q*-value = .039 and .039, respectively; Data Set S2).

### Differential analysis of microbial abundance between groups

The analysis of bacterial phyla abundance revealed no statistically significant differences between the three groups (Data Set S1). Furthermore, the ratio of Firmicutes to Bacteroides (F/B ratio) was not altered in *RLS* and *INS* compared to *CTRL* (Data Set S1).

**Figure 2:**
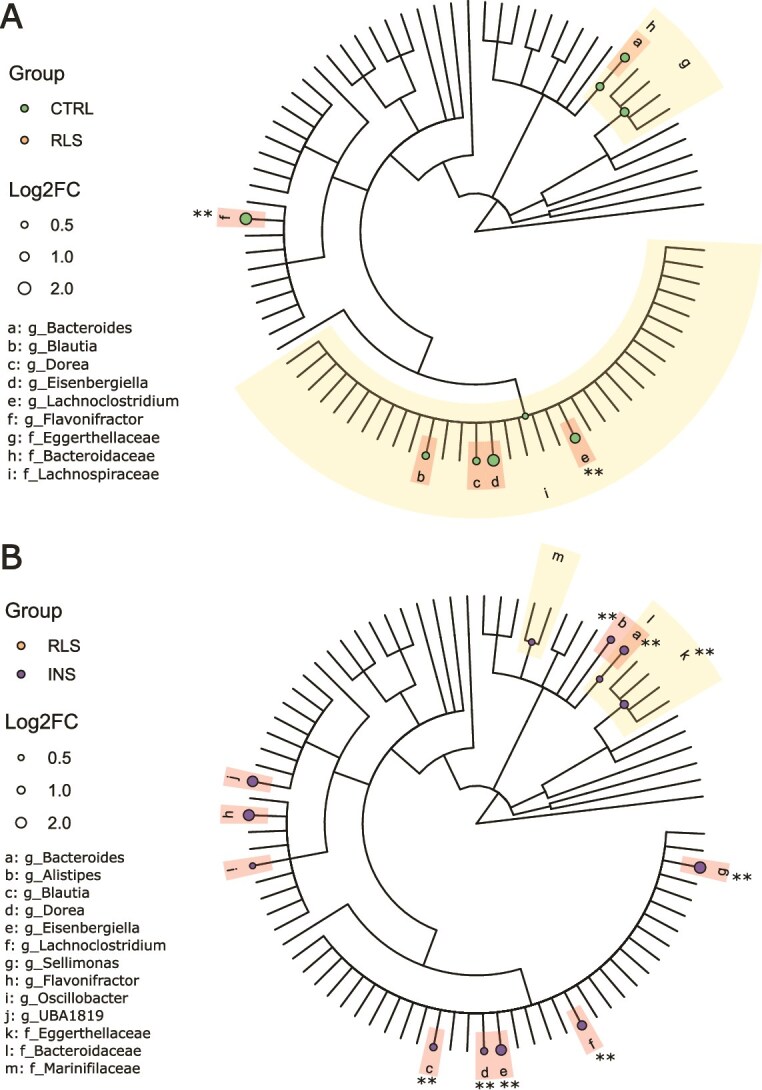
General overview of taxonomic differences in fecal microbiota compositions between patients with Restless Legs Syndrome patients (*RLS*), insomnia (*INS*), and healthy controls (*CTRL*). Circular phylogenetic tree (cladogram) showing differing taxa at family and genus level in the comparisons (**Panel A**) *RLS* vs. *CTRL* and (**Panel B**) *RLS* vs. *INS*, identified by Deseq2 analysis. The legends shown on the left side of each panel indicate the microbial families (f_) and genera (g_) corresponding to the labelled branches, as well as the proportional size of the dots according to the Log2 fold change (Log2FC). Statistical test: DESeq2 pipeline—Wald test, correcting for age, sex, BMI, sequence batch, and presence of mood disorders. Families and genera showing a BH-adjusted *p*-value (*q*-value) < .05 are indicated with two asterisks (**); families and genera showing a trend (nominal *p*-value < .05) are indicated by shaded areas surrounding the corresponding branches. The dots legend indicates the group with an increased abundance of the selected taxa.

At the family level, no statistically significant abundance differences (*q*-value < .05) were found in the comparison of *RLS* to *CTRL,* although *Bacteroidaceae*, *Eggerthellaceae*, and *Lachnospiraceae* families displayed a trend towards decrease in RLS compared to CTRL (*p*-value <.05). *Eggerthellaceae* was significantly reduced in *RLS* versus *INS* (*q*-value = .015; Figure 2 and Data Set S1), while *Bacteroidaceae* and *Marinifilaceae* showed a trend toward decrease. No significant alterations were found in the comparison between *INS* and *CTRL* (Data Set S1).

**Figure 3:**
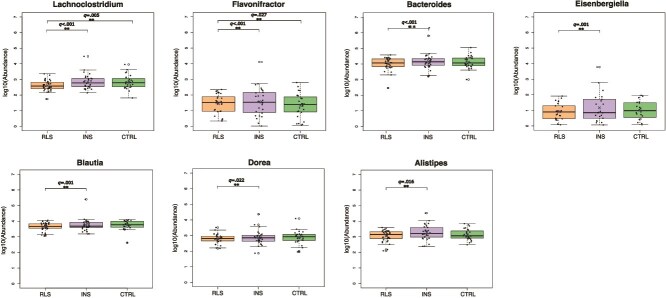
Specific taxonomic differences in fecal microbiota compositions between patients with Restless Legs Syndrome patients (*RLS*), insomnia (*INS*), and healthy controls (*CTRL*). Boxplots show the microbial genus abundance in *CTRL*, *RLS*, and *INS* groups. Sample size *n* = 94. The central line indicates the median; the *X* indicates the mean; the box represents the interquartile range (IQR), and the whiskers extend to 1.5 × IQR. Black dots represent individual samples; white circles are outliers. Statistical test: DESeq2 pipeline—Wald test, correcting for age, sex, BMI, sequence batch, and presence of mood disorders. ^**^ indicates BH-adjusted *p*-value (*q*-value) < .05. Reference group in all comparisons is on the left.

Importantly, we identified two genera that were significantly decreased in *RLS* compared to *CTRL*, i.e. *Lachnoclostridium* (*q*-value = .005) and *Flavonifractor* (*q*-value = .027)*,* with four additional genera showing a trend of change in the same direction (*Bacteroides, Eisenbergiella*, *Blautia*, and *Dorea*). The same six genera were significantly decreased in *RLS* compared to *INS* (Figure 3 and Data Set S1) while, importantly, none of them was significantly altered or showed a trend in the *INS* versus *CTRL* comparison (Data Set S1).

To confirm the above-described results, we used an alternative analytical pipeline as described in the Materials and Methods section. This second analysis confirmed a trend toward decrease of *Lachnoclostridium*, *Flavonifractor*, and *Bacterioides* genera in the *RLS* versus *CTRL* comparison, although not reaching statistical significance (Data Set S3).

Finally, we used our dataset to predict functional abundance. The analysis returned 323 predicted pathways. After BH correction for multiple testing, no pathway resulted significantly enriched in RLS compared to INS or CTRL (Data Set S1).

### Genera abundance variations according to clinical features and treatment

Microbial abundance at the genus level was analyzed according to clinical parameters available for *RLS* participants (Data Set S2). Significant associations were found with IRLS score, with *Clostridium sensu stricto 1* and *Terrisporobacter* showing a negative association (*q*-value < .001 and *q*-value= .048, respectively) and *[Eubacterium] brachy group* showing a positive association (*q*-value = .030). We also found a positive association with the PSQI for *Faecalibacterium* (*q*-value = .008)*.* No significant results emerged with other RLS clinical parameters, mood comorbidities, ISI and BDI-II scores (Data Sets S3 and S4). Focusing on genera altered in the comparison between the *RLS* and *CTRL* group (*Lachnoclostridium, Flavonifractor, Bacteroides, Eisenbergiella,* Blautia, and *Dorea*), *Lachnoclostridium* showed a trend towards a decrease in patients with longer disease duration and with motor phenotype.

Finally, genera abundance was analyzed according to pharmacological treatments in *RLS.*

*RLS* treated with α2δ-ligands displayed increased levels of *Enterorhabdus* (*q*-value < .001) (Data Set S2), but they did not show differences in the six genera that emerged from the comparison between the *RLS* and *CTRL*. Conversely, patients under dopamine agonists treatment showed a significant decrease in the genus *Eisenbergiella* (*q*-value = .001) (Data Set S2).

## Discussion

To the best of our knowledge, this is the first study describing the gut microbiome landscape in RLS, identifying a distinct microbial signature compared to INS patients and healthy controls. Only two previous studies [19,20] have reported the presence of SIBO and dysbiosis in RLS patients, but the first relied on the breath test and the second included only a very small sample size. We did not observe significant differences in alpha and beta diversity between *RLS, INS*, and *CTRL*, although alpha diversity tended to be higher in the *RLS* group compared to *CTRL*. This increase was significantly higher in *RLS* patients with longer disease duration. This finding is somewhat unexpected, as a decrease in alpha diversity has been described in several diseases [16,33,34] and high-quality sleep is generally associated with its increase. However, a recent study found that alpha diversity increased with disease duration in narcolepsy type 1 patients [35], suggesting that, similarly to what was observed in our cohort, gut microbiota diversity may grow throughout time in chronic diseases. We also found that beta diversity was significantly higher in patients with longer disease duration, further supporting a remodeling of gut microbiota over the course of the disease. Alpha diversity tended to be higher in the *RLS* patients without sensory symptoms, although it did not reach statistical significance; one could speculate that this increase might play a protective role against developing a more severe phenotype. In our study, phyla abundance and F/B ratio did not differ between the three groups. On the other hand, we identified significant differences for *Lachnoclostridium* and *Flavonifractor* genera that were specific to *RLS* patients. In addition, the abundance of the *Lachnospiraceae* family, to which *Lachnoclostridium* genus belongs, showed a trend toward decrease in *RLS* patients. A reduction of the *Lachnospiraceae* family has also been reported in other neurological diseases such as Alzheimer’s disease, mild cognitive impairment [36,], and Parkinson’s disease [37]. Furthermore, alterations in the abundance of the *Lachnospiraceae* family and its genera have previously been linked to sleep disorders, in particular INS [18,38,39]. However, there is considerable variability in these findings, particularly regarding the taxa involved and the direction of change, likely due to differences in analytical and statistical approaches, including the choice of covariates. Importantly, the taxonomic differences in fecal microbiota observed between *RLS* patients and *CTRL* were largely unaffected by dopaminergic treatment within the *RLS* group. The only genus significantly altered by dopaminergic therapy was *Eisenbergiella*, which showed a trend towards decrease in *RLS* respect to *CTRL*.

Overall, the differences discussed above between *RLS* and *CTRL* were not observed in patients with INS, suggesting that these taxa may be specific to *RLS*. Therefore, while these findings indicate a specificity for RLS, they also leave open the possibility that some of these differences may be associated with specific clinical features of *RLS*. We found that *Lachnoclostridium* genus tended to decrease in RLS patients with longer disease duration and in those who did not report sensory symptoms. Alterations in the abundance of the *Lachnospiraceae* family and genera belonging to it, in particular *Lachnoclostridium* and *Blautia*, have been previously linked to sleep-related variables. Smith et al. [40], analyzing 26 participants, including only males, screened for the absence of gastrointestinal illness, found that the abundance of the *Lachnospiraceae* family and *Blautia* genus negatively correlated with sleep efficiency and total sleep time, assessed using actigraphy. However, their study also noted that other two members of the *Lachnospiraceae* family were positively associated with these parameters, highlighting the potential for genus-level variability and sex-specific effects. Another study, including 72 INS patients, reported a positive association between *Lachnoclostridium* abundance and sleep efficiency [38].

Although functional analysis in our dataset did not return significant findings, it is worth noting that the members of the *Lachnospiraceae* family could play a role in gut and CNS homeostasis and exert anti-inflammatory effects through the production of short-chain fatty acids (SCFAs). SCFAs are metabolites produced in the colon through the fermentation of dietary fibers and starch; they contribute to strengthening the intestinal epithelial barrier and regulating the production of antimicrobial peptides [41]. At the same time, SCFAs can regulate circadian rhythms by influencing the expression of peripheral [42] and central [43] circadian clock genes. They may also cross the blood–brain barrier via the transcellular lipophilic pathway, potentially contributing to the circadian fluctuation of symptoms observed in RLS patients [44].

There is also growing interest in the role of systemic inflammation in the pathophysiology of RLS as highlighted by several studies that showed: (1) a frequent association of RLS with inflammatory and infectious conditions [7], (2) the elevation of levels of cytokines in the blood [10], and (3) alterations of inflammatory networks in proteomic and transcriptomic studies [5,6]. Another potential pathogenic factor inserted in this network could be hepcidin, a hormone that regulates iron homeostasis and may act as a link between inflammation and iron deficiency in RLS [45]. In fact, a reduction in SCFA levels allows pro-inflammatory substances, such as lipopolysaccharides present in the intestinal lumen, to enter the bloodstream. These substances increase systemic inflammation and stimulate the production of hepcidin, which binds to ferroportin and promotes its degradation, reducing iron absorption from the gastrointestinal tract, and its availability to the CNS [46].

In our cohort, one-third of *RLS* reported gastrointestinal symptoms, most of which involved the upper digestive tract, with a prevalence higher than that observed in *CTRL* but not significantly different from that of *INS*. Only 3 (8 per cent) *RLS* had IBS (vs. 28 per cent, 9/32, RLS patients [19]), while none of the *CTRL* or *INS* patients presented IBS (vs. 4 per cent, 1/25, general population controls [19]). Moreover, we did not observe cases of Crohn’s disease or Celiac disease, a finding consistent with previous reports showing that RLS is more prevalent among patients with these autoimmune disorders, but not vice versa [47]. Taken together, these findings support the hypothesis that dysbiosis in RLS is an independent factor from gastrointestinal comorbidities. In inflammatory bowel diseases, the higher prevalence of RLS may be explained by the combination of multiple precipitating factors (e.g. systemic immune activation, iron deficiency), with dysbiosis possibly acting as an additional element that helps cross the symptomatic threshold. Overall, our results highlight the role of dysbiosis as a microenvironmental factor that may contribute to the pathogenesis of RLS in genetically predisposed individuals, and which should be investigated regardless of gastrointestinal comorbidities or symptoms. This point is particularly relevant, as it has already been demonstrated that in patients with SIBO and RLS, treatment of SIBO with rifaximin led to an improvement of RLS symptoms [48, 49]. Moreover, a gluten-free diet has also been shown to improve RLS symptoms, further supporting the role of gut-related factors in RLS and Celiac disease [47].

In conclusion, our study is the first to thoroughly assess the gut microbiota composition in patients with RLS. Among its strengths are: (1) the use of two control groups, INS patients, and healthy subjects, which allowed us to identify taxa altered in RLS compared to both groups, but not in INS patients compared to healthy controls. These taxa may therefore represent an RLS-specific metagenomic signature; (2) the inclusion of multiple covariates (age, sex, BMI, and presence of mood disorders), which are known to affect microbiota composition, thus confirming that the observed differences in taxa abundance are independent of these confounding factors; and (3) the use of multiple statistical approaches to evaluate the reproducibility of the results.

However, our study has several limitations, including a small sample size, the lack of detailed dietary assessments, and a highly ethnically homogeneous population. To address this concern, we included control groups with the same characteristics. However, this highly selected RLS population limits the generalizability of the results, as the microbiota composition may be influenced by genetic background [50]. Another limitation is that we did not consider other comorbidities beyond gastrointestinal and mood disorders, nor concomitant medications except those specifically prescribed for RLS and INS. This decision was driven by the primary aim of the study, which was to focus on factors already known to influence the gut microbiota, and by the relatively small sample size, which did not allow robust subgroup analyses.

This research could potentially contribute to a more comprehensive understanding of the RLS pathogenesis, facilitate more accurate diagnoses, and help identify targeted treatments. However, it is necessary to confirm these findings by increasing the sample size, including patients from different ethnicities, and including follow-up data. It is also important to correlate microbiota data with other laboratory data, such as blood inflammation markers and metabolomic analysis, to qualitatively and quantitatively assess SCFAs, as well as instrumental findings, such as actigraphy data.

## Supplementary Material

Supplementary_File_Datasets_zsaf383

## Data Availability

The data underlying this article are available in the article. Analysis pipeline used is available on github (https://github.com/LabBrainAgeing/iRLS_Metagenomic).
